# Microbial larvicides for malaria control in The Gambia

**DOI:** 10.1186/1475-2875-6-76

**Published:** 2007-06-07

**Authors:** Silas Majambere, Steven W Lindsay, Clare Green, Balla Kandeh, Ulrike Fillinger

**Affiliations:** 1 School of Biological and Biomedical Sciences, Durham University, Durham, DH1 3LE, UK; 2 Medical Research Council Laboratories, Fajara, The Gambia; 3 National Malaria Control Programme, Banjul, The Gambia

## Abstract

**Background:**

Mosquito larval control may prove to be an effective tool for incorporating into integrated vector management (IVM) strategies for reducing malaria transmission. Here the efficacy of microbial larvicides against *Anopheles gambiae *s.l. was tested in preparation for a large-scale larviciding programme in The Gambia.

**Methods:**

The impact of water-dispersible (WDG) and corn granule (CG) formulations of commercial *Bacillus sphaericus *strain 2362 (*Bs*; VectoLex^®^) and *Bacillus thuringiensis *var.*israelensis *strain AM65-52 (*Bti; *VectoBac^®^) on larval development were tested under laboratory and field conditions to (1) identify the susceptibility of local vectors, (2) evaluate the residual effect and re-treatment intervals, (3) test the effectiveness of the microbials under operational application conditions and (4) develop a method for large-scale application.

**Results:**

The major malaria vectors were highly susceptible to both microbials. The lethal concentration (LC) to kill 95% of third instar larvae of *Anopheles gambiae s.s*. after 24 hours was 0.023 mg/l (14.9 BsITU/l) for *Bs *WDG and 0.132 mg/l (396 ITU/l) for *Bti *WDG. In general *Bs *had little residual effect under field conditions even when the application rate was 200 times greater than the LC_95_. However, there was a residual effect up to 10 days in standardized field tests implemented during the dry season. Both microbials achieved 100% mortality of larvae 24–48 hours post-application but late instar larvae were detected 4 days after treatment. Pupae development was reduced by 94% (95% Confidence Interval = 90.8–97.5%) at weekly re-treatment intervals. Field tests showed that *Bs *had no residual activity against anopheline larvae. Both microbials provided complete protection when applied weekly. The basic training of personnel in identification of habitats, calibration of application equipment and active larviciding proved to be successful and achieved full coverage and control of mosquito larvae for three months under fully operational conditions.

**Conclusion:**

Environmentally safe microbial larvicides can significantly reduce larval abundance in the natural habitats of The Gambia and could be a useful tool for inclusion in an IVM programme. The costs of the intervention in this setting could be reduced with formulations that provide a greater residual effect.

## Background

At the start of the new millennium malaria is still deeply entrenched in Africa and effective malaria control is under threat from drug and insecticide resistance [1,2]. In response to that, mosquito larval control has recently received renewed attention by the international scientific community [3-11] and recent attempts to develop integrated vector management (IVM) strategies for different eco-epidemiological settings re-consider mosquito larval control as one of the tools to reduce malaria transmission [12].

Promising new formulations of the microbial larvicides *Bacillus sphaericus *(*Bs*) and *B. thuringiensis var. israelensis *(*Bti*) have recently been shown to give excellent control of the major vectors of malaria in Africa [4,13]. Use of these biological control agents is better than chemical larvicides since they are very species specific, environmentally safe [14] and appear not to induce resistance when used together [15]. It is envisaged that the utilization of such biological control agents may be best carried out using a vertical approach that actively involves local communities [10,16].

The national strategy for malaria control in The Gambia includes larval control [17], yet there has been no detailed evaluation of this methodology. Whilst *Bs *and *Bti *have been tested in different ecological settings in Africa [4,18-22], the riparian habitats found in The Gambia represent a novel habitat for investigating these microbials. In the presented study the efficacy of microbial larvicides was tested against malaria vectors in The Gambia, West Africa, to identify the optimal formulations, dosages and application methods in order to prepare for a large-scale larviciding programme.

## Methods

### Study area

The Gambia is in the southern Sahel and is characterized by a single rainy season from June to October. The country lies in an area of open flat Sudan savannah that is dominated by the River Gambia, a large, slow moving waterway, characterized by tidal movements and saltwater intrusions as far as 200 km up river. River Gambia is representative of many large river systems in Africa. Its tidal movements flood successive belts of vegetation from the mangrove forest through flooded *Phragmites*, sedge and grass species, punctuated by large bands of barren floodplain. The tidal movement of the river and its flooding during the rainy season creates suitable breeding habitats for malaria vectors [23,24].

**Figure 1:**
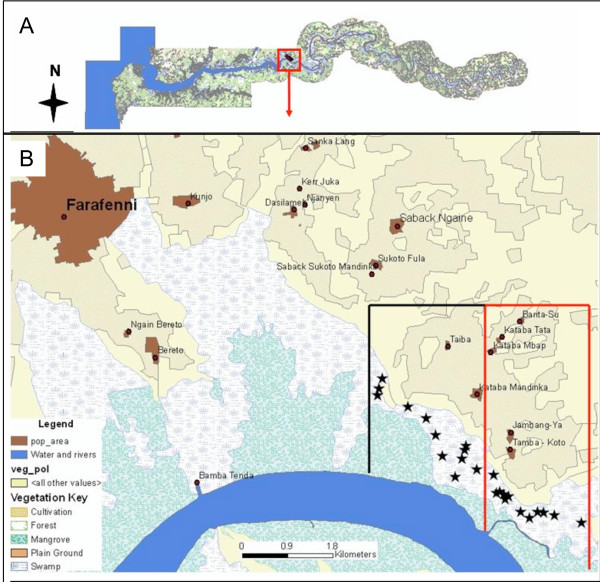
Map of The Gambia, West Africa (A) and the study area (B). The black line encloses the control, the red line the intervention area. The 24 sentinel sites for larval surveys are marked as stars.

The study was based in and around Farafenni town (UTM zone 28 1500200mN, 435500mE), in the central part of the country, about 100 km from the coast (Figure 1). Laboratory and standardized field tests were carried out at Farafenni Field Station of the Medical Research Council (MRC) Laboratories. Field tests were implemented near Tamba-Koto village, 10 km east of Farafenni. The area is predominantly flat farmland and woodland savannah. The main inland crops are sorghum, millet, groundnut and pumpkin and in the floodplains swamps rice is grown during the rainy season. The villages in the area are discrete clusters of houses and are not scattered as seen in many parts of Africa. The primary malaria vectors are *Anopheles gambiae sensu stricto, Anopheles arabiensis *and *Anopheles melas *[23,25].

### Climate

Data on daily minimum and maximum temperatures were available from the meteorological station at Kaur 30 km from Farafenni town. Rainfall was collected with a rain gauge at the MRC station, Farafenni.

### Larvicides

Water-dispersible granular formulations (WG/WDG) of the commercial strains of *Bs *(VectoLex^® ^strain 2362, Lot number 115-498-PG, 650 International Toxic Units, ITU/mg) and *Bti *(VectoBac^® ^strain AM65-52; Lot number 114-114-32, 3000 ITU/mg; Valent BioSciences Corporation, Illinois, USA,) were tested in the laboratory and under field conditions, in a similar manner to that described by Fillinger *et al*. [13] in Kenya, in order to make direct comparisons between west and east Africa. WG/WDG formulations were applied as liquid with handheld or knapsack sprayers. *Bs *(VectoLex^®^, Lot number 117-999-NB, 50 ITU/mg) and Bti (VectoBac^®^, Lot number 131-661-NB, 200 ITU/mg) corn granule (CG) for hand application or motorized granule spreaders was evaluated under field conditions only.

### Laboratory assays

Laboratory assays were conducted to assess the susceptibility of the principal malaria vector in The Gambia, *An. gambiae s.s*., to microbial larvicides. Laboratory assays were carried out with a colony of insectary-reared larvae originated derived from wild-caught mosquitoes collected from Saruja in The Gambia and maintained at the MRC Laboratory in Farafenni since 2002. All mosquito larvae used in the laboratory experiments were reared at a room temperature of 28°C, 80% relative humidity and an approximate 12 hour light : 12 hour dark cycle. Larvae were reared in transparent, 1.5 L capacity plastic containers (24 × 17 × 8 cm) filled with 1 L tap water that had been left in the insectary for at least 48 hours to equilibrate. Larvae were fed by adding a pinch of crushed Tetramin^® ^(Tetra, Germany) fish food spread evenly on the water surface twice daily.

Assays were performed with the WG/WDG formulation of VectoLex^® ^and VectoBac^® ^to determine their minimum effective dosages following the standard testing procedures for microbial tests [14]. Fifty third instar larvae were randomly collected for the experiment from several bowls to compensate for size differences that could have reflected the amount of food available [26] and transferred to new 1.5 L plastic containers filled with 1 L of the test solution or distilled water only (control). On every test date a fresh stock solution of 100 mg/l WG/WDG was prepared and test aliquots made up to 1 L with distilled water. After range finding tests [14], five to six different test concentrations were chosen for each experiment. Test concentrations ranged between 0.001 and 0.1 p.p.m for *Bs *and between 0.001 and 0.016 p.p.m for *Bti*. Each experiment contained an untreated control. The experiment was run in three replicates at the same time and the entire experiment carried out on five occasions. Larvae were not fed during the experiments and all tests were run at ambient temperature ranging between 21 and 34°C. Larvae were counted and mortality scored after 24 hours. Where mortality exceeded 10% in the controls, the experiment was discarded and repeated.

### Standardized field trials

Standardized field trials were conducted at the MRC field station in Farafenni during the rainy (September to October 2004) and dry season (December 2004 to May 2005) to identify the optimum dosages of *Bs *and *Bti *required under field conditions and to evaluate the residual effect and re-treatment intervals for the test microbials. Artificial ponds were created following the experimental design of Fillinger *et al*. [13]. Eighteen light blue plastic tubs (0.5 m diameter) were buried into an open sunlit field in three lines of six tubs (distances between tubs was approximately 2 m). The tubs were filled with approximately 6 kg of top soil from the experimental area to provide the abiotic and biotic conditions suitable for mosquitoes. Tubs were filled with tap water and maintained at a depth of 0.4 m. Overflow holes were created at the 0.4 m level and screened with nylon netting to allow excess water to leave the tubs during heavy rainfall and prevent larvae from being washed over the edges. The habitats were left open for mosquito oviposition. Experiments were implemented eight to nine days after the tubs were set-up to allow third and fourth instar larvae to develop. Water temperatures during the experiments ranged between a minimum of 23°C and a maximum of 40°C. Acknowledging the hazard artificially created breeding sites present, all habitats were carefully screened for pupae twice daily with a dipper and visually and any pupae were removed to prevent the emergence of malaria vectors.

**Figure 2:**
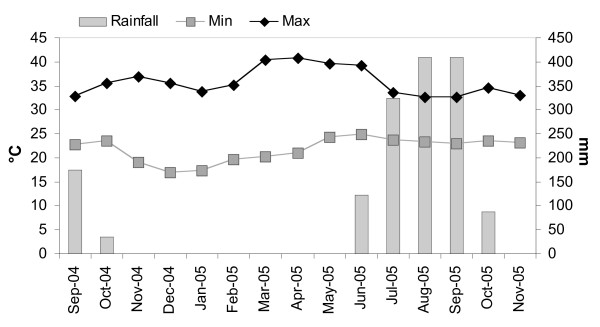
Temperature (°C) and rainfall pattern (mm) during the study period.

Of the 18 artificial habitats, six served as untreated controls and two treatments (six tubs each) were allocated to the remaining 12. Treatment and control ponds were selected randomly using a web-based randomisation tool [27]. Treatment concentrations were calculated on the basis of a standard water depth of 0.1 m and fixed surface area [28,29] irrespective of the actual water depth to simulate operational procedures. Both microbial larvicides were tested in this set up at the following concentrations: *Bs *WDG at 0.5, 1.0, 2.5 and 5 mg/l (0.5, 1, 2.5 and 5 kg/ha), and, *Bti *WG at 0.2 mg/l (equivalent to a surface application of 0.2 kg/ha). Each concentration was tested in six habitats at a time and repeated once (i.e. 12 habitats in total). The first round of tests with *Bs *were implemented during the rainy season 2004 and replicated during the dry season 2005. *Bti *was tested during the cold dry season, in December, and repeated during the hot dry season in May (Figure 2). Liquid formulations were sprayed evenly over the entire water surface of the habitats using a 250 ml handheld sprayer. Each day the average number of larvae and pupae per dip (350 ml capacity dipper, Clarke Mosquito Control Products, Illinois, USA) was determined by taking five dips from four different directions of each pond close to the edge and one from the middle. Mosquito larvae were classified as anophelines or culicines and recorded as early (1^st ^and 2^nd^) or late (3^rd ^and 4^th^) instars. After counting, larvae were returned to the water and pupae removed. Treatment was done once at day 0. The experiment was terminated when the difference between late instar and pupae density was no longer statistically significant between control and treatment tubs. A sub-sample of 69 *Anopheles *adults were allowed to emerge from pupae collected from the control and identified morphologically; rDNA-PCR markers were used for species determination of adults of the *An. gambiae *species complex [30].

### Field trial

Based on the results from the laboratory and the standardized field tests a pilot-scale field operation was designed and implemented between August and November 2005 to test the efficiency and life span of the larvicides under natural conditions in representative habitat types in the floodplains of the River Gambia. The field tests served to identify (1) the operational requirements e.g. time needed per surface area treated, equipment and manpower needed, (2) the optimal microbial and (3) the best formulation in preparation for large-scale larviciding campaigns scheduled for the following rainy season 2006.

**Figure 3:**
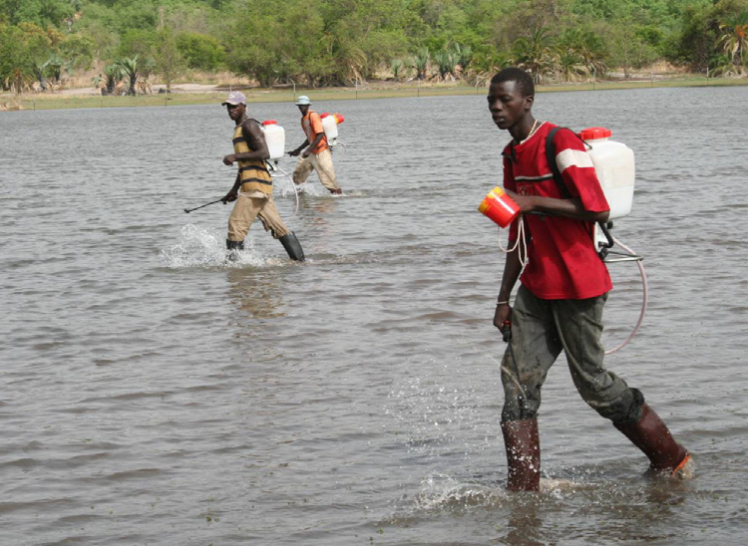
Liquid application of microbial larvicides with 15 litres capacity knapsack sprayers on open water surface (edge of floodwater).

**Figure 4:**
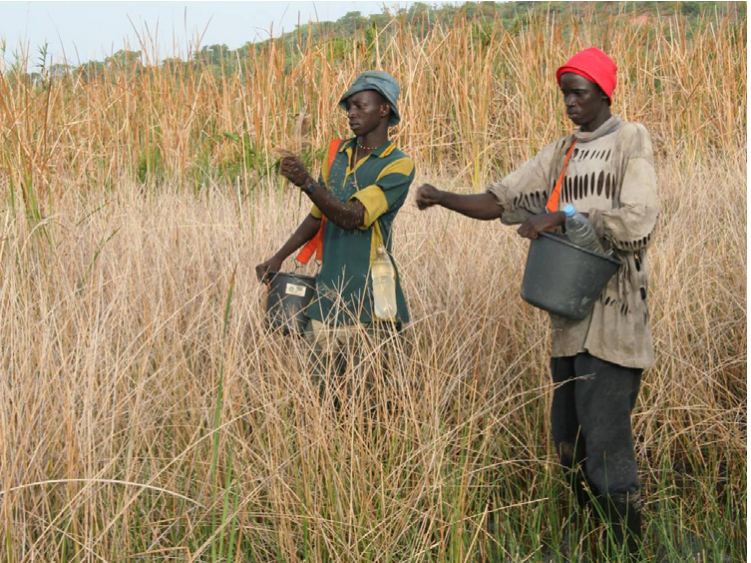
Hand application of corn granule in highly vegetated areas of the floodplains.

Liquid (WDG) and granule (CG) formulations of both *Bs *and *Bti *were tested. Liquids were applied using 5 L capacity compression sprayers (Mesto Resistent No. 3600, Freiberg, Germany) or 15 L capacity diaphragm knapsack sprayers (SOLO^® ^475, Sindelfingen, Germany; Figure 3). Both sprayers were operated at an average pressure of 4 bar. Corn granules were either applied by hand carrying the granules in a 5 L bucket on a carrying strap over the shoulder (Figure 4) or were spread with 13 L backpack power chemical applicators (MD 150DX-13 Maruyama, Tokyo, Japan) covering a swath width of 10–15 metres.

The pilot zone was situated 10 km east of Farafenni and had an area of 24 km^2^. The area included the major breeding habitats for anophelines in this region of The Gambia: extensive rice fields, pools that were people-made and natural, and large floodwater areas interspersed with grass. Notably, aquatic habitats harbouring anopheline larvae in the Gambia might be described as 'atypical' when compared with other parts of Africa. The habitats are water-fed primarily through the flooding of the river and are additionally under tidal influence leading to flooding and contraction of the habitats which are usually shallow but can be extensive in size (Figure 3) and are probably fairly typical for many large rivers in the Sahel.

After mapping all aquatic habitats in the pilot zone the area was divided into an intervention and a non-intervention zone (Figure 1). In the intervention zone, 6 km^2 ^was routinely larvicided. In each zone 12 sentinel sites were randomly selected from the total list of habitats for measuring mosquito larval density. The sentinel sites were located in rice fields and floodwater habitats covered with grass and sedge. Larviciding was implemented under operational conditions by a team of four men from the National Malaria Control Programme who had undergone two weeks of training prior to the field trial. The monitoring of the intervention's impact in the 24 sentinel sites was implemented independently by the research team. The larviciding teams were unaware of the location of the sentinel sites.

*Bs *treatments were applied at rates of 1 kg/ha for WDG and 15 kg/ha for CG; dosages proven to be effective from the standardized field trials and previous experiences [4,13]. *Bs *WDG was tested for two consecutive weeks and followed by *Bs *CG for one week. This allowed the authors to train larviciding staff how to use different application equipment and assess whether the two formulations performed differently under field conditions. Larval density was surveyed using the standard dipping technique [31]. Ten dips were taken at each sentinel site to determine the larval density at the day of the first treatment (day 0) and at day two, four and seven after treatment for three consecutive weeks. Purposive sampling was done to maximise the sensitivity of collections. Re-treatments took place on a weekly basis if late instar larvae occurred at day four.

Following the *Bs *field test, operational application of *Bti *was evaluated at dosages of 4.0 kg/ha for CG for two weeks, followed by WDG applications of 0.2 kg/ha for seven weeks. Dosages were based on laboratory and field trial results and on previous studies [4,13]. During the application of *Bti *larval density was monitored once a week in the sentinel sites using the same methodology as described above. The monitoring was implemented one to three days after application.

Due to the specific habitat characteristics in the tidal floodwater of The River Gambia we covered the entire surface of all aquatic habitats with larvicide.

### Analyses

LC_50 _and LC_99 _values were determined using log-probit regression analysis. The percentage reduction in larval mosquito densities in the standardized field trials was calculated using the formula of Mulla *et al*. [32]: % Reduction = 100 - (C_1_/T_1 _× T_2_/C_2_) ×100, where C_1 _and C_2 _describe the average number of larvae in the control tubs pre- and post-treatment, T_1 _and T_2 _describe the average number of larvae in the treated tubs pre- and post-treatment. Mean number of larvae and pupae per dip in control and treatment sites in field tests were compared using non-parametric Mann-Whitney tests. The tests were implemented separately for each sampling day comparing mean numbers of immature stages in the controls with treatments. When multiple comparisons of more than one treatment and control were made the Bonferroni correction was used to define the alpha cut off value. The corrected significance levels are presented with the figures. All analyses were carried out using version 11.0 of the SPSS statistical software package.

### Ethics

Ethical approval for this study was given by the Joint Gambian Government and Medical Research Council's Laboratories in The Gambia, as well as Durham University's Ethics Advisory Committee.

## Results

### Climate

Figure 2 summarizes average minimum and maximum temperatures and the monthly rainfall during the study period from September 2004 to November 2005. The dry season extended from November 2004 to May 2005 and can be portioned into a 'cold dry season', from November to February, and a 'hot dry season', from March to May. The rainy season is characterized by more constant temperatures with little difference between minimum and maximum values. Rain fell only once during the experiments on day 4 of the rainy season test of low (0.5 and 1 kg/ha) *Bs *WDG dosages, but did not appear to influence the results.

### Laboratory assays

After 24 hours exposure of third instar larvae of *An. gambiae s.s*. to *Bs *WDG (VectoLex^®^, 650 BsITU/mg), a concentration of 0.004 mg/l (2.6 BsITU/l) caused 50% mortality (LC_50_) and a concentration of 0.023 mg/l (14.9 BsITU/l) caused 95% mortality (LC_95_). *Bti *WDG (VectoBac^®^, 3000 ITU/mg) concentrations of 0.039 mg/l (117 ITU/l) killed 50% of the larvae and 0.132 mg/l (396 ITU/l) 95% (Table 1).

**Table 1:** Laboratory bioassays results of *Bs *and *Bti *WDG/WG against third instar larvae of *Anopheles gambiae s.s*. after 24 h exposure (lethal concentrations (LC) in p.p.m.)

WDG/WG Formulations	LC_50 _(95% CI)	LC_95 _(95% CI)	Slope (SE)	c^2 ^(d.f.)
VectoLex (650 BsITU/mg)	0.004 (0.003<LC<0.005)	0.023 (0.016<LC<0.042)	2.208 (0.112)	123.518 (25)
VectoBac (3000 ITU/mg)	0.039 (0.033<LC<0.047)	0.132 (0.100<LC<0.199)	3.110 (0.141)	140.513 (23)

CI, confidence interval; SE, standard error; d.f., degrees of freedom

### Standardized field trials

Throughout the year, oviposition occurred soon after the artificial habitats were set up and immature stages of anopheline and culicine mosquitoes detected after four to five days. Overall *Anopheles *larvae accounted for 40% of larvae collected during the trials. 69 *Anopheles *adults that emerged from pupae collected from the control tubs were identified to species level. 36 *Anopheles *adults belonged to the *An. gambiae *s.l. species complex and PCR analyses revealed that the tubs contained a mix of *An. arabiensis *(66%), *An. gambiae *s.s. (30%) and *An. melas *(4%). Since there were no differences of the impact of the larvicides on anophelines and culicines in the standardized field trials the data were pooled for all analyses and presentation.

#### Bti WG

**Figure 5:**
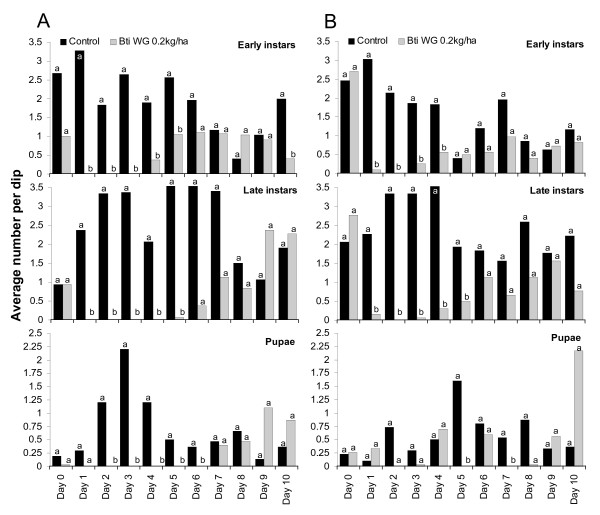
Impact of *Bti *WDG at 0.2 kg/ha on early and late larval and pupae density in standardized field tests. A: during cold dry season (Dec); B: during hot dry season (May). Daily differences in immature densities were analysed using Mann-Whitney tests at a significance level of p < 0.05. Different letters (a, b) on bars indicate a significant difference at the specific sampling day.

Field trials with *Bti *WG were implemented with the minimum dosage [33] required to cause 100% mortality within 24–48 hours after application as identified in the laboratory assays. Since no improvement of the impact or activation of any residual effect was expected [34] higher dosages were not tested after the minimum dosage of 0.2 kg/ha under standardized field conditions killed all larvae within 48 hours and provided therefore optimum control for the period of one week (Figure 5 and Table 2). Although reduced late instar densities were recorded up to eight to ten days after application (Table 2) these differences were only statistically significant up to day five (Figure 5) in both test periods. Late instar larvae and pupae developed in increasing numbers five to six days after *Bti *application. The seasons had little impact on the outcome of the trials. Notably, pupal production could not be completely suppressed despite the well-controlled implementation of the experiment, although pupal production was more successfully suppressed during the cold than the hot dry season. The results indicate that weekly treatment intervals can reduce pupae production by 64–94%. A recent study though showed that higher rates of the WG formulation of *Bti *may produce longer control since the WG particles redistribute throughout the water column after application (S. Krause, personal communication) and, therefore, the effect of higher *Bti *WG rates on field residual control of *An. gambiae *requires further study.

**Table 2:** Percent reduction (%) of late instar larvae (*Anopheles *and culicine combined) after application of *Bti *WDG at 0.2 kg/ha in the cold (Dec) and hot (May) dry season

Day after application	Cold dry season	Hot dry season
1	100	95
2	100	100
3	100	95
4	100	94
5	98	81
6	90	54
7	67	68
8	45	68
9	0	33
10	0	74

#### Bs WDG

**Figure 6:**
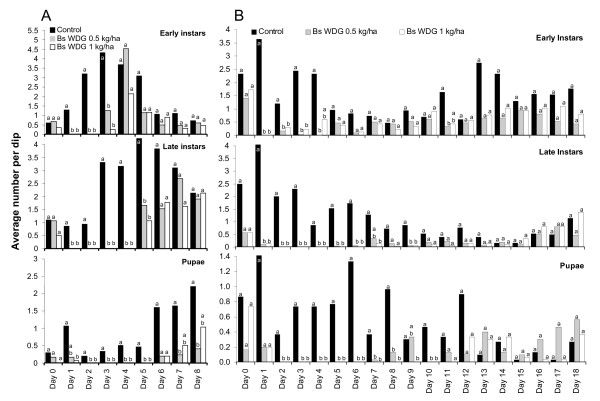
Impact of low dosages of *Bs *WDG (0.5 and 1 kg/ha) on immature mosquito density in standardized field tests. A: during rainy season; B: during dry season. Daily differences in immature densities were analysed using Mann-Whitney tests at a significance level of p < 0.017. Different letters (a, b) on bars indicate a significant difference at the specific sampling day.

**Figure 7:**
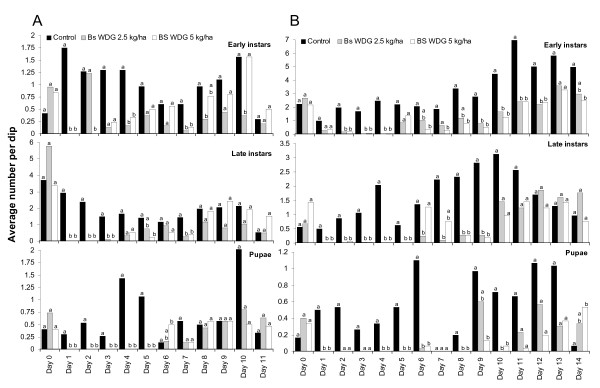
Impact of high dosages of *Bs *WDG (2.5 and 5 kg/ha) on immature mosquito density in standardized field tests. A: during rainy season; B: during dry season. Daily differences in immature densities were analysed using Mann-Whitney tests at a significance level of p < 0.017. Different letters (a, b) on bars indicate a significant difference at the specific sampling day.

Four different doses of *Bs *WDG were tested (0.5, 1.0, 2.5 and 5.0 kg/ha) and each experiment run twice to evaluate whether any residual effect of the larvicide could be detected which would allow extended re-treatment intervals. The results of the impact of the different dosages are presented in Figure 6 and 7. The results are shown separately for the replicates implemented during the rainy (A) and the dry season (B). The daily percent reduction of late instar larvae is summarized in Table 3.

**Table 3:** Percent reduction (%) of late instar larvae (Anopheles and culicine combined) after application of *Bs *WDG in different dosages in the dry and rainy season

	Rainy season				Dry season			
	
Day after application	0.5 kg/ha	1.0 kg/ha	2.5 kg/ha	5.0 kg/ha	0.5 kg/ha	1.0 kg/ha	2.5 kg/ha	5.0 kg/ha
1	100	100	100	100	96	96	100	100
2	100	100	96	100	100	100	100	95
3	100	100	89	68	100	100	100	100
4	100	100	68	85	100	100	100	100
5	60	44	52	55	100	100	88	100
6	59	0	86	69	76	100	96	60
7	11	0	62	2	4	68	89	84
8	9	0	74	0	40	100	91	94
9	-	-	70	1	54	54	58	97
10	-	-	36	0	0	17	60	86
11	-	-	-	-	0	0	4	75
12	-	-	-	-	0	48	0	70
13	-	-	-	-	0	0	0	54
14	-	-	-	-	0	0	-	66
15	-	-	-	-	0	0	-	-
16	-	-	-	-	0	0	-	-
17	-	-	-	-	0	0	-	-
18	-	-	-	-	0	0	-	-

-, test was terminated earlier

Irrespective of dosage and season 96–100% larval mortality was achieved 24–48 hours after application. No residual effect of a single *Bs *application was detected during the rainy season at any application rate tested but was extended during the dry season for all tested dosages (Figure 5 and 6, Table 3). Statistically significant reductions in pupae densities were achieved up to five days post-treatment in the rainy season and up to 10 days during the dry season. There were no statistically significant differences between the different test concentrations (Figure 6 and 7). Consequently, pupae development could be reduced by over 95% when *Bs *WDG was applied at weekly intervals. Low dosages have shown to be as effective as high dosages. During the dry season similar suppression of pupae densities could be achieved at 10-day re-treatment intervals.

### Field trial

Field trials were conducted in the floodplains of the River Gambia to confirm results from standardized field set up and to evaluate the effect of larviciding under operational conditions. The field tests were implemented during the rainy season which is the main malaria transmission season in The Gambia and the period with most larval habitats. At the start of the field trials late instar *Anopheles *larvae were found in 33% of all sentinel sites. The proportion of habitats with late instar *Anopheles *increased in the non-intervention sites with continuous rainfall to 67% in October 2005. Culicine and anopheline larvae co-existed in most of the habitats and did not show any difference in response to the larviciding. Both sub-families have, therefore, been pooled for presentation and analyses.

**Figure 8:**
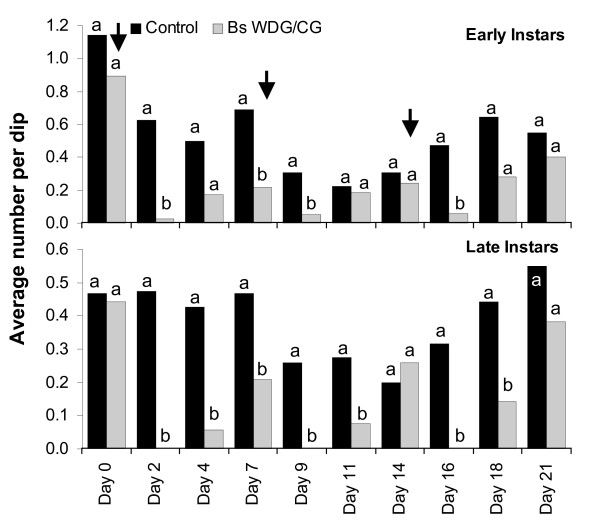
Efficiency of *Bs *treatments under operational field conditions. *Bs *application took place on day 0, day 7 and day 14 (arrows). WDG formulation was applied on day 0 and 7; CG formulation was applied on day 14. Differences in immature densities were analysed using Mann-Whitney tests at a significance level of p < 0.05. Different letters (a, b) on bars indicate a significant difference at the specific sampling date.

**Figure 9:**
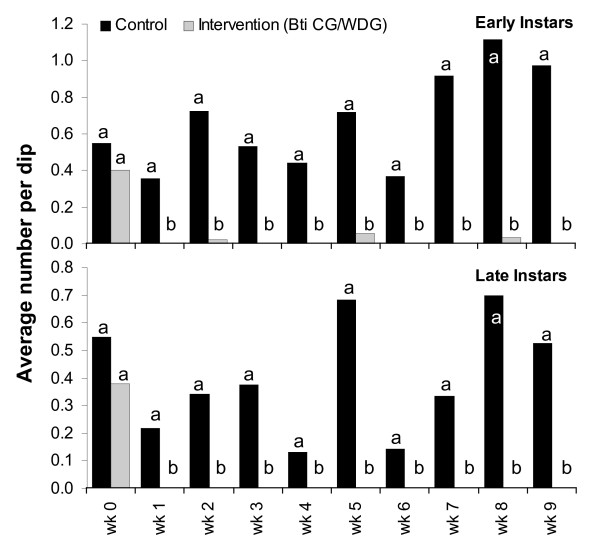
Efficiency of *Bti *treatments under operational field conditions. *Bti *application took place weekly. The monitoring of the sentinel sites was done 1–3 days after application. CG formulation was applied in week (wk) 1, 2 and 3, WDG formulation was applied from week 4. Differences in immature densities were analysed using Mann-Whitney tests at a significance level of p < 0.05. Different letters (a, b) on bars indicate a significant difference at the specific sampling date.

*Bs *WDG and CG formulations were evaluated to detect any residual effect of the microbial under operational application in the field. Application took place at weekly intervals to evaluate whether continuous application might result in an increasing residual effect with time. The results of the three week trial are presented in Figure 8. 100% mortality of late instar larvae was achieved two days post-treatment at any application date irrespective of the formulation applied. A residual effect of the microbial which would allow re-treatment intervals greater than one week was not detected (Figure 8), which supports the results from the standardized field trials. Weekly application of *Bti *under operational conditions (Figure 9) achieved a consistent suppression of larval development over the entire nine weeks study period with minimum dosages (as identified in laboratory) irrespective of the formulation and equipment used. Surprisingly, pupae were not collected under field conditions in either the intervention or control sites.

Larvicides were applied by four men from 7:00 to 13:00 (6 hours) a day. While staff worked continuously to cover the entire study area, each habitat was spayed only once a week. All formulations could be successfully applied under operational conditions and were equally effective. Different application equipment though had an impact on the time required per surface area treated. On average seven hectares were treated per day (0.29 ha/person/hour) using 5 L compression sprayers or 13 L motorised granule spreaders; nine hectares were covered using 15 L knapsack sprayers (0.38 ha/person/hour) and 5 hectares when granules were applied by hand (0.21 ha/person/hour).

## Discussion

The results show that the major malaria vectors in The Gambia are highly susceptible to *Bs *and *Bti *under laboratory and field conditions, with *Bs *even more toxic per weight applied than *Bti*. The LC values found in the laboratory experiments are very similar to those found in earlier studies [13,35] conducted in East Africa suggesting that the susceptibility of malaria vectors to microbial larvicides is inherent to the species and not to the ecological settings of the area. *Bs *has shown residual activity for two to 10 weeks in previous studies [36-39], with repeated applications increasing the likelihood of greater residual activity [4,40]. Larvicides with long residual activity would be advantageous for larviciding campaigns because less manpower and larvicide would be required, helping to keep down costs and increase effectiveness. However, in contrast to previous results *Bs *did not show extended residual effect under field conditions in The Gambia even after repeated treatments and when the application rate was as high as 200 times the LC_95 _(5 kg/ha). Only a slightly extended residual effect up to 10 days could be detected in the standardized field tests implemented during the dry season (January to March) but not the wet season. It can be hypothesized that the different daily water temperature profile in the experimental tubs during the rainy season might be responsible for the reduced effect of the microbial. Although the average air temperatures did not differ between the experimental periods in the rainy and dry seasons the low variation between minimum and maximum temperatures during the rainy season (Figure 2) will have resulted in high water temperatures for longer during the rains compared with the dry season. High water temperatures result in faster destruction of the protein toxin [41]. The low residual activity could also be due to the low larval density observed in the artificial and natural habitats. *Bs *seems to persist or recycle in some environments because it rapidly increases its numbers in the midgut of killed larvae [42-44]. Where larval densities were high the residual activity of the microbial larvicides appears to be greater [45,46]. Dead and dying larvae release the bacteria into the water increasing the bacterial content of the water and infecting new generations of larvae.

The presence and abundance of pupae can serve as a proxy measure for adult mosquito emergence, since pupae survive for only a few days before adult emergence. The identification of the most productive habitats for adults could help target larviciding operations especially in the extensive water surface areas of the river's floodplains. In this study no pupae were collected in the field during the pilot field tests. This unexpected finding may be a consequence of the dipping technique. Although the technique is commonly used for studying larval ecology in Africa, it appears to be inappropriate for sampling the very sensitive and agile pupae from natural aquatic habitats, particularly in The Gambia where larval densities are generally low consequently leading to even lower pupae densities. This insensitivity of the sampling technique is further compounded by the highly aggregated distribution of pupae in natural habitats compared to larvae [31,47,48]. Even in the tubs dipping underestimated the density of pupae. Sweep nets may prove to be a better sampling tool since they collect 10 times more pupae in fewer sweeps than dips (see also [47]). Since pupae abundance is often used for establishing the 'productivity' of habitats [8] further studies to develop more efficient sampling protocols are desirable.

Large-scale application of larvicides in The Gambia will be implemented during the rainy season when the river floods and surface water is plentiful. At the end of the wet season over 90% of the *Anopheles*-containing breeding sites dry up quickly leaving only a few dry season refugia. For these dry season sites a targeted application of *Bs *might be useful to suppress the build up of the adult population at the start of the rains. The results indicate that with commercially available microbials weekly larviciding will be necessary during the rainy season in The Gambia. In this instance the use of *Bti *products is preferred since the costs for this microbial are far lower than *Bs *[4] and the development of resistance is unlikely [15,34,49]. Very low dosages of 0.2 kg/ha (representing the LC_99_) lead to optimal suppression of mosquito larvae and pupae which is consistent with results from East Africa [4,13].

Granule and liquid formulations have proven equally effective in killing mosquito larvae but the selection of application equipment was important for the speed of coverage. The 5 L compression sprayers were easier to carry than the higher capacity sprayers, but they were slower to use because they needed to be refilled more frequently. This was exacerbated by the fact that most water bodies in the floodplains of The Gambia are very shallow and muddy, and, therefore, unsuitable for water collection, which led to long distances being covered to re-fill the sprayers. Another disadvantage of the compression sprayers was that they have to be pressurized by pumping air into the tank before spraying which proved difficult on the very muddy ground in the floodplains. The problem of finding suitable water sources for mixing WDG formulations and the increasing plant growth during the rainy season favours the application of granule formulations in an environment like the river floodplains. Motorized granule spreaders provided excellent coverage and proved especially useful in areas with tall vegetation where access on foot is impossible. However, when filled with the microbials they weighed close to 20 kg and walking on soft ground became difficult and, coupled with the loud noise of the engine, made them uncomfortable for long-term use. The relatively high purchase and running costs of motorized spreaders (approximately $300/spreader plus fuel costs) compared to knapsack sprayers (approximately $100/sprayer) represent another disadvantage in resource poor African settings. Based on the pilot field trial, it is recommended for a large-scale larviciding programme in The Gambia to use 15 L knapsack sprayers for all large, open water surface areas, and to use granule formulations for highly vegetated areas. In the view of the authors hand application is the preferred method for larvicide application because it represents a low-tech and low-cost technology. Even though granule distribution does not result in an even application, as that achieved with motorized sprayers, it is easily manageable and maintenance free. Nevertheless, motorized sprayers must be used when tall vegetation dominates or access on foot is impossible due to high water level or soft underground.

The basic training of larviciding personnel in identification of habitats, calibration of application equipment and active larviciding proved to be successful and achieved full coverage and control of mosquitoes for three months under fully operational conditions. To reduce labour and management effort though it would be desirable to have larvicides available which would express extended efficiency under extreme climate conditions. Microbial larvicides were chosen in this study because, in contrast to many other larval control agents, they exhibit the highest environmental safety to non-target organisms and application personnel, they are very easy to handle and are unlikely to lead to the development of resistance [15,34,49,50]. Nevertheless, it would be useful to explore whether greater persistence could be achieved with alternative products. Organophosphates, like temephos, appear to be less useful since they rarely show much persistence compared with microbials [9,51]. Moreover, organophosphates can have a negative impact on non-target organisms [52,53] and need careful resistance management. On the other hand, the use of insect growth regulators (IGRs), like pyriproxyfen, might prove more advantageous [54-56]. IGRs have been highly successful elsewhere when applied at monthly intervals, although this was usually administered in highly confined habitats [56,57]. Whether this residual effect could be replicated in a highly mobile aquatic environment like the floodplains of The Gambia needs careful evaluation. The greatest disadvantage of IGRs though is the difficulty in monitoring whether they are still effective or not since larvae will always be detected in the water and the development and emergence of pupae needs to be observed, which represents a challenge given the difficulty of collecting any pupae. Moreover complicated monitoring systems using emergence cages or similar devices might not be easy to handle in a large-scale operational programme.

## Conclusion

The results support the hypothesis that the implementation of large-scale larviciding with commercially available microbials in The Gambia will lead to a reduction in larval abundance in the natural habitats. Both microbial strains tested, can be applied successfully in extended floodplain areas either as liquid with knapsack sprayers or as granules by hand and motorised sprayers. Due to the lack of residual effect of *Bs*, products *Bti *should be applied weekly during the rainy season. Dry season refugia should be targeted with bi-monthly *Bs *applications.

Environmentally safe microbial larvicides could be an additional tool in an IVM programme in The Gambia but due to the lack of residual effect of the microbial larvicides, there is a need to assess the costs of weekly applications in consideration of reduction in transmission intensity.

## Authors' contributions

UF, SWL and SM designed the study. SM was responsible for the implementation of the study in the laboratory and the field. CG carried out the species identification of members of the *An. gambiae *complex. BK assisted with the organization of the field trial. UF and SM analysed the data. All authors were involved in the manuscript writing, read and approved the final manuscript.
